# Asymmetry of Deep Medullary Veins on Susceptibility Weighted MRI in Patients with Acute MCA Stroke Is Associated with Poor Outcome

**DOI:** 10.1371/journal.pone.0120801

**Published:** 2015-04-07

**Authors:** Johanna Mucke, Markus Möhlenbruch, Philipp Kickingereder, Pascal J. Kieslich, Philipp Bäumer, Christoph Gumbinger, Jan Purrucker, Sibu Mundiyanapurath, Heinz-Peter Schlemmer, Martin Bendszus, Alexander Radbruch

**Affiliations:** 1 University of Heidelberg, Department of Neuroradiology, INF 400, 69120 Heidelberg, Germany; 2 University of Mannheim, Department of Psychology, Schloss Ehrenhof Ost, 68131 Mannheim, Germany; 3 University of Heidelberg, Department of Neurology, INF 400, 69120 Heidelberg, Germany; 4 German Cancer Research Center (DKFZ), Department of Radiology, INF 280, 69120 Heidelberg, Germany; University Medical Center (UMC) Utrecht, NETHERLANDS

## Abstract

**Background and Purpose:**

Due to its sensitivity to deoxyhemoglobin, susceptibility weighted imaging (SWI) enables the visualization of deep medullary veins (DMV) in patients with acute stroke, which are difficult to depict under physiological circumstances. This study assesses the asymmetric appearance of prominent DMV as an independent predictor for stroke severity and outcome.

**Materials and Methods:**

SWI of 86 patients with acute middle cerebral artery (MCA) stroke were included. A scoring system from 0 (no visible DMV) to 3 (very prominent DMV) was applied for both hemispheres separately. A difference of scores between ipsi- and contralateral side was defined as asymmetric (AMV+). Occurrence of AMV+ was correlated with the National Institute of Health Stroke Scale (NIHSS) Score on admission and discharge, as well as the modified Rankin Scale (mRS) at discharge. Ordinal regression analysis was used to evaluate NIHSS and mRS as predictors of stroke severity, clinical course of disease and outcome.

**Results:**

55 patients displayed AMV+ while 31 did not show an asymmetry (AMV–). Median NIHSS on admission was 17 (11–21) in the AMV+ group and 9 (5–15) in the AMV– group (p = 0.001). On discharge median NIHSS was 11 (5–20) for AMV+ and 5 (2–14) for AMV– (p = 0.005). The median mRS at discharge was 4 (3–5) in the AMV+ group and 3 (1–4) in AMV– (p = 0.001). Odds ratio was 3.19 (95% CI: 1.24–8.21) for AMV+ to achieve a higher mRS than AMV– (p = 0.016).

**Conclusion:**

The asymmetric appearance of DMV on SWI is a fast and easily evaluable parameter for the prediction of stroke severity and can be used as an additional imaging parameter in patients with acute MCA stroke.

## Introduction

Magnetic resonance imaging (MRI) is increasingly used in the diagnosis of acute ischemic stroke since it enables not only estimation of the infarct core and penumbra but also displays other parameters such as the assessment of thrombus length and location.[1] Nevertheless, additional prognostic imaging parameters may be valuable for the assessment of initial stroke severity and the estimation of clinical outcome.[2]

Deep medullary veins (DMV) are located in the periventricular white matter as long and tiny vessels, serving as drainage for the white matter of the cerebral hemispheres. They run perpendicular to the long axis of the lateral ventricle and converge into collecting trunks which drain into the subependymal veins.[3] Owing to their small caliber, visualization of normal DMV is usually not possible with conventional imaging techniques and has only been reported in a few cases.[4] Pathologic DMV can be distinguished from normal DMV due to earlier visibility by contrast-enhanced imaging, an increased caliber and length, an irregular course with frequent changes of diameter and a tendency to present in clusters.[3] Abnormal DMV are associated with several cerebral pathologies such as malignancies, venous thrombosis and arteriovenous malformations.[4] Furthermore, increased DMV have been reported on susceptibility weighted imaging (SWI) in the area of ischemic infarction in children and in patients with Sturge-Weber syndrome.[5]

SWI is a high resolution 3D phase enhanced gradient echo method (GRE) that is highly sensitive to susceptibility differences of adjacent tissues. The SWI technique converts the GRE-phase images, which reflect the magnetic field distortion resulting from susceptibility changes, to a special phase mask and combines this mask with the corresponding GRE magnitude image. Thereby, contrast and conspicuity of susceptibility inclusions, such as deoxyhemoglobin or calcium and thus MR contrast is increased.[6–8] Due to the additional phase information venous blood, haemorrhage, iron storage, and calcium can be imaged more accurately compared to conventional T2*-weighted GRE-magnitude images.[9–11]

SWI enables depiction of normal, hypointense DMV in several patients; however impeccable image quality is essential. Artifacts resulting from patient motion, a low signal-to-noise ratio and increased susceptibility changes at air-parenchyma interfaces often impede the depiction of physiological DMV.[12] Therefore, the sole appearance of DMV cannot be used as an imaging marker for a pathologic process. In contrast, an asymmetric appearance of DMV in the affected hemisphere with a difference in caliber of DMV might indicate a pathological finding in acute ischemia, since the DMV in acute stroke should present prominent due to the increase of deoxyhemoglobin.

In the current study, we assessed the frequency of abnormal, asymmetric DMV patterns in patients with acute ischemic stroke and correlated the findings with the initial NIHSS status, stroke severity and outcome.

## Methods

### Patients

This retrospective study was approved by the local ethics committee of the University of Heidelberg (“Ethikkommission Medizinische Fakultät Heidelberg”, S-330/2012). Due to the retrospective nature of this study and the poor clinical condition of the majority of the included patients the ethics committee did not require informed written consent of the included patients. A total of 96 patients with an acute middle cerebral artery (MCA) stroke who underwent MRI including SWI at the University Hospital of Heidelberg Medical Center between September 2009 and August 2013 were eligible. Three patients had to be excluded due to major motion artifacts and in seven patients either the NIHSS or mRS score was missing, leaving a total of 86 patients (41 male (47.7%), 45 female (52.3%)) for study analysis. In 27 patients the exact onset of stroke symptoms was known while 59 patients were wake-up strokes. Stroke severity and outcome was assessed by using the National Institutes of Health Stroke Scale (NIHSS) [13] by a neurologist with NIHSS-certification. All patients had an initial NIHSS score >2. For all patients NIHSS score was determined at both the time of admission and discharge. The modified Rankin Scale (mRS) [14]was determined at discharge as an indicator of stroke outcome. We additionally assessed the success of endovascular treatment in all 22 patients who received mechanical thrombectomy. Treatment success was defined as full vessel recanalization based on digital subtraction angiography images using the TICI score (thrombolysis in cerebral ischemia, TICI 2b or 3).

### Image Acquisition

Images were acquired during the clinical workup using a 3 Tesla MR system (Magnetom Tim Trio or Verio Siemens Healthcare, Erlangen, Germany, with identical technical parameters) with a 12-channel head-matrix coil. SWI data were collected with a 3D, fully flow-compensated GRE sequence using the following parameters: echo time (TE) = 19.7 ms, repetition time (TR) = 27 ms, 48 slices, flip angle (FA) = 15°, bandwidth (BW) = 140 Hz/px, FoV = 172 x 230, acquisition matrix = 216 x 320, and voxel size = 0.72 x 0.72 mm x 3 mm; no interpolation was applied. Parallel imaging (generalized autocalibrating partially parallel acquisitions, GRAPPA) with an acceleration factor of 2 and 24 reference lines resulted in a total acquisition time of 2:16 min.

### Image analysis

**Fig 1:**
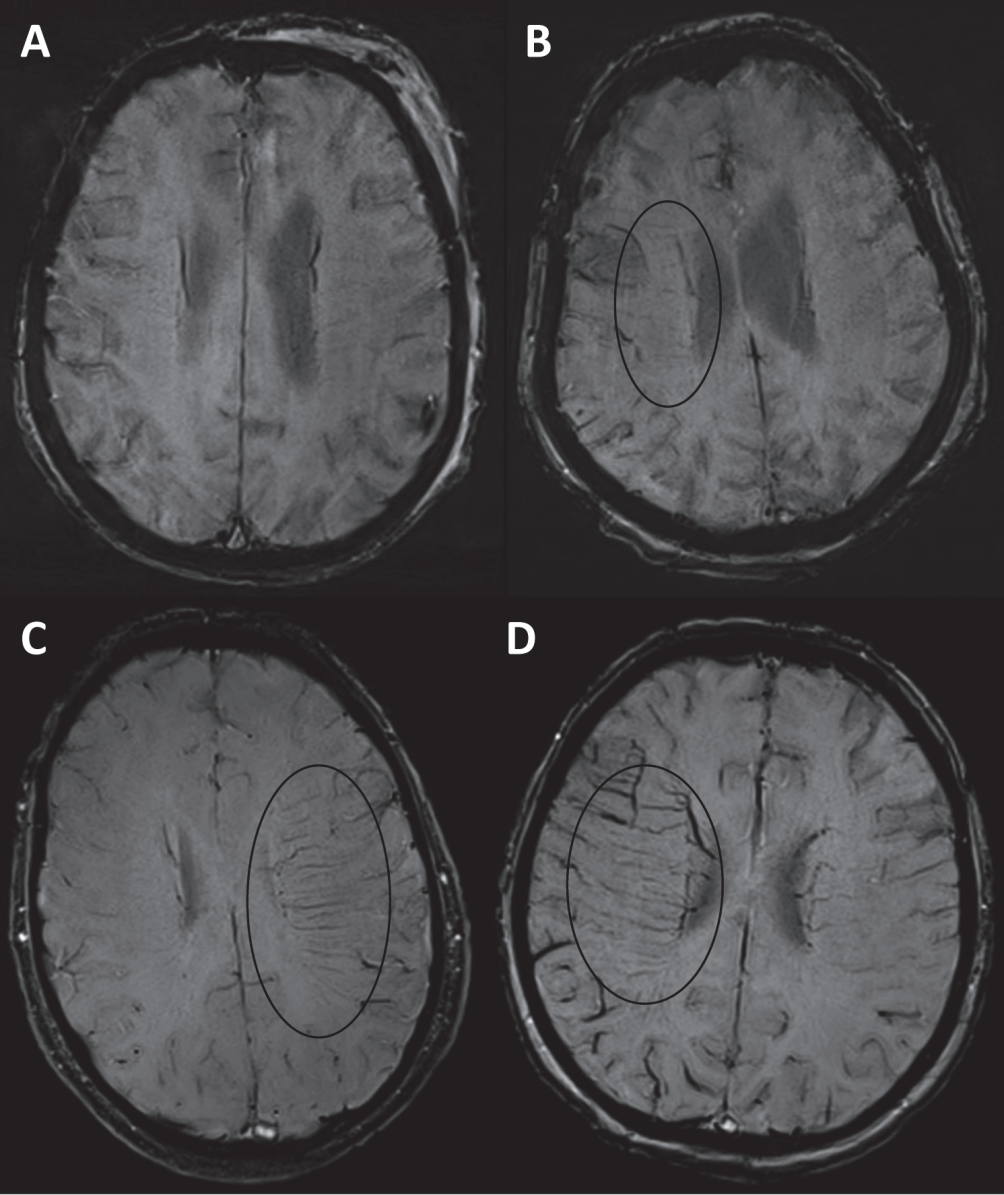
Scoring system for the quantification of DMV. A) 0 = no visible DMV B) 1 = faintly visible DMV C) 2 = unequivocal visible DMV D) 3 = very prominent DMV.

**Fig 2:**
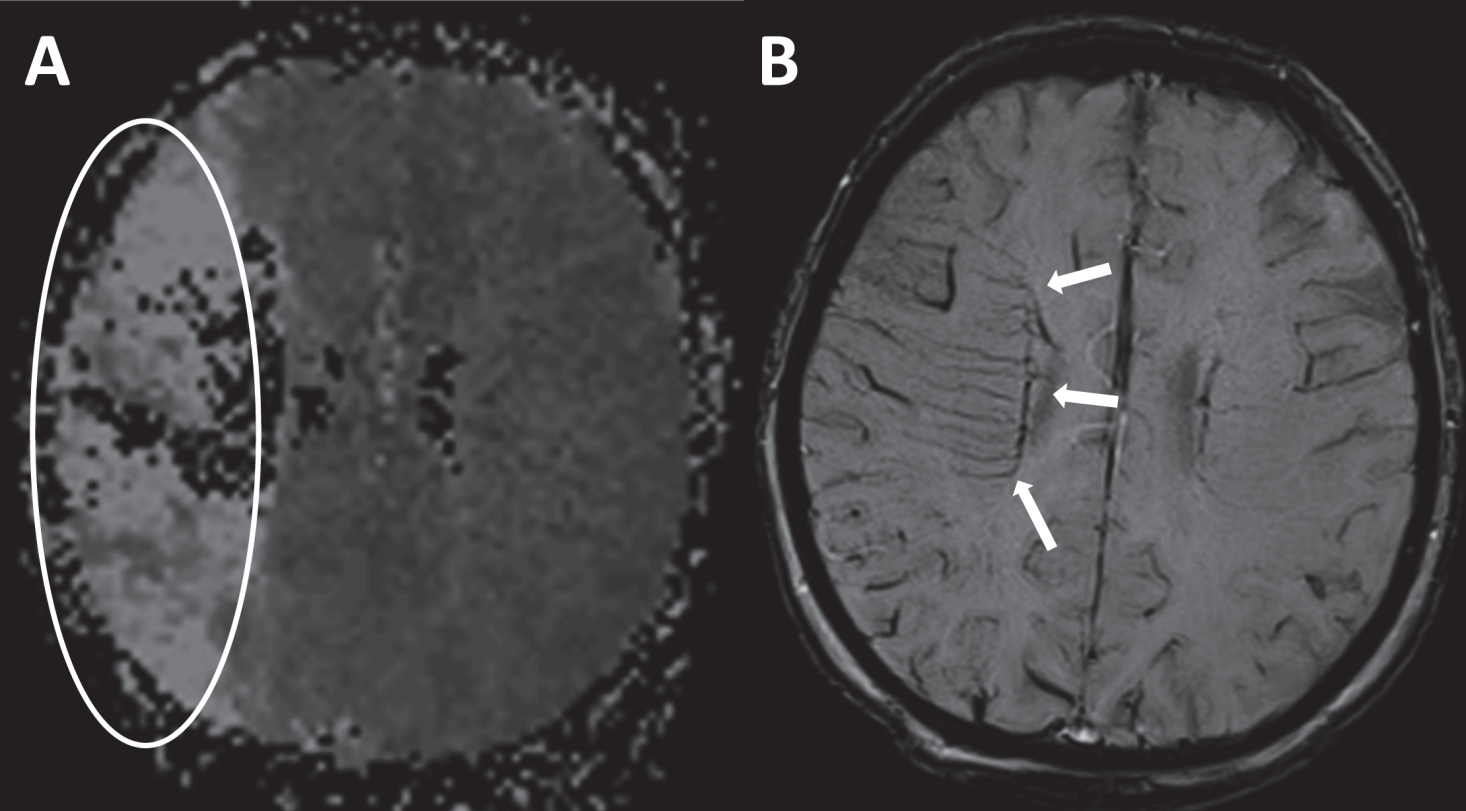
Patient with right MCA infarction. A) Reduced perfusion within the territory of the right MCA. B) Asymmetric appearance of prominent periventricular DMV within the infarcted area (arrows).

The SWI sequences of all patients were assessed with regard to presence of DMV by two readers (JM and AR), based on visual inspection. Each hemisphere was evaluated separately using a scoring system from score 0 to 3 to quantify visibility and prominence of the DMV. A score of 0 was defined as not visible, a score of 1 indicated a faint visibility, a score of 2 indicated unequivocal visibility, a score of 3 was assigned to very prominent DMV (Fig 1). The score of the unaffected, contralateral hemisphere was deducted from the one of the affected hemisphere. A difference of the scores was defined as an asymmetry of DMV (AMV+), whereas an equal score for both hemispheres was evaluated as no asymmetry of DMV (AMV–) (Fig 2). Additionally, thrombus size was determined on SWI images by measuring the length of the hypointense susceptibility vessel sign.[1] Diffusion-perfusion mismatch was evaluated by visual assessment, comparing the area of reduced diffusion on diffusion weighted images (DWI) with the area of impaired perfusion on the time-to-peak-perfusion map. Mismatch was defined as a focal perfusion reduction which exceeded the extent of the lesion on DWI by 20%.[15, 16]

### Statistical analysis

Statistical analyses were carried out using IBM SPSS statistics 21 at a significance level of α = 0.05. For continuous scales such as time between onset of symptoms and MRI, period of hospitalization and thrombus size values were given as mean ± standard deviation (SD) and Student’s t-test for independent samples was applied for statistical analyses. In case of ordinal scales of NIHSS and mRS, data are presented as median and 1^st^ quartile- 3^rd^ quartile. A Chi-square-test was used to compare the frequency of treatment success, relevant mismatch and lethality in both groups. NIHSS and mRS scores in AMV+ and AMV– groups at admission and discharge were compared using the Mann-Whitney-U-Test. To evaluate the correlation of AMV and clinical course and outcome of patients, proportional odds logistic regression was performed and results were expressed in odds ratios with 95% confidence intervals (CI).[17] Based on former studies, the NIHSS was clustered according to 5-point divisions (0–5, 6–10, 11–15, 16–20, 21–25, >25).[18, 19] Deaths were scored 42 on NIHSS and assigned to group 6 of the clustered NIHSS. For evaluation of the patients’ clinical course logistic regression was performed on the difference of grouped NIHSS on admission and discharge (Δ NIHSS) while outcome was assessed using the mRS in its original design for logistic regression. We adjusted regression analyses for NIHSS on admission using its grouped design for assessment of the clinical course and the original baseline score for analysis of the patients’ outcome. Furthermore we adjusted for age, presence of wake-up stroke and duration of hospitalization. Baseline stroke severity and age were proven to be the most important prognostic factors [20, 21, 22, 23] and the presence of wakeup strokes and the duration of hospitalization as the time point of testing mRS and NIHSS on discharge were considered most influential on outcome parameters in this study. The interrater reliability was assessed by use of the intraclass correlation coefficient (ICC) with a two-way mixed model (absolute agreement).

## Results

Fifty-five patients (45.5% male, 54.5% female) presented an asymmetry of the deep medullary veins (AMV+) whereas 31 (51.6% male, 48.4% female) did not show any presence of DMV at all or a symmetrical visibility of DMV in both hemispheres (AMV–). All patients rated as AMV+ were scored higher on the affected hemisphere than on the non-affected hemisphere. Interrater reliability was good for both right and left hemisphere with an ICC of 0.88 and 0.86 respectively and the resulting difference between both sides (0.88) (all p<0.0001). In no case the two readers disagreed about presence or absence of AMV. (S1 and S2 Tables)

Median NIHSS scores were significantly higher in the AMV+ group both on admission (17.00 (11.00–21.00)) and discharge (11.00 (5.00–20.00)) compared to the AMV– group with a median score of 9.00 (5.00–15.00) on admission (p = 0.001) and 5.00 (2.00–14.00) on discharge (p = 0.005). Also, median mRS score at discharge for AMV+ patients was significantly higher (4 (3–5)), than in AMV– (3 (1–4) (p = 0.001)). The odds ratio for patients with AMV+ to have a worse outcome in terms of higher mRS scores was 3.19 (95% CI: 1.24–8.21) (p = 0.016). Mortality was 9.1% (5 patients) in AMV+ versus 0% in AMV– (χ² = 3.0, df = 1, p = 0.08). (S3 and S4 Tables)

Ordinal regression analysis did not reveal a significant difference of ΔNIHSS (admission and discharge) between AMV+ and AMV– groups in the clinical course (p = 0.07). Also, there was no difference between the frequency of wake up stroke (63.6% in AMV+ vs. 77.4% in AMV–; χ² = 1.75, df = 1, p = 0.19) and the time between onset of symptoms and MRI (3.9 ± 1.7 h in AMV+ vs. 4.1 ± 1.3 in AMV–; p = 0.70) in patients with known onset of symptoms. (S5 Table)

Neither age (69.3 ± 14.5 years in AMV+ vs. 69.4 ± 17.3 years in AMV–; p = 0.98), nor period of hospitalization (6.0 ± 3.9 days for AMV+ and 5.5 ± 3.1 days for AMV–; p = 0.56), presence of a diffusion-perfusion mismatch (63.6% in AMV+ vs 54.8% in AMV–; p = 0.38) or thrombus size (11.7 ± 6.5 mm in AMV+ vs. 7.8 ± 4.5 mm in AMV–; p = 0.05) differed significantly between the two groups. (S6 Table) Clinical and demographic features of all patients and results are summarized in Table 1.

**Table 1:** Clinical and demographic features of AMV+ and AMV–.

	AMV + (n = 55)	AMV– (n = 31)	p-values
Demographic data			
Male, n (%)	25 (45.5)	16 (51.6)	
Female, n (%)	30 (54.5)	15 (48.4)	
Age, mean ± SD	69.3 **±** 14.5	69.4 **±** 17.3	0.98
NIHSS score, median (Q1-Q3)			
Admission	17 (11–21)	9 (5–15)	**0.001**
Discharge	11 (5–20)	5 (2–14)	**0.005**
Modified Rankin Scale, median (Q1-Q3)	4 (3–5)	3 (1–4)	**0.001**
Lethality, n (%)	5 (9.1)	0 (0)	0.08
Time between onset of symptoms and MRI (hours)	3.9 ± 1.7	4.1 ± 1.3	0.70
Wake-up strokes, n (%)	35 (63.6)	24 (77.4)	0.19
Mismatch on PWI-DWI, n (%)	35 (63.6)	17 (54.8)	0.38
Thrombus length (mm)	11.7 ± 6.5	7.8 ± 4.5	0.05
Period of hospitalization (days)	6.0 ± 3.9	5.5 ± 3.1	0.56

Forty-one of the included 86 patients received treatment either in terms of intravenous thrombolysis 19 patients), endovascular treatment (6 patients) or a combination of both approaches (16 patients). Twenty-six (63.4%) of all 41 treated patients were successfully treated defined as radiologically proven recanalization regardless of clinical features in case of endovascular treatment, or clinical improvement if treated with intravenous thrombolysis only. Of the 19 patients (13 AMV+, 6 AMV–) who were solely treated with intravenous rtPA, the success rate was 46.2% in AMV+ and 66.7% in AMV– (χ² = 0.69, df = 1, p = 0.41). Endovascular treatment alone was successful in 75.0% of AMV+ patients and in 0% of cases with AMV– (χ² = 3.0, df = 1, p = 0.08) while recanalization rate of patients who received intravenous lysis before the endovascular intervention was 78.6% in AMV+ and 100% in AMV– (χ² = 0.52, df = 1, p = 0.47). None of the differences in treatment outcome were statistically significant. Hence no correlation between treatment outcome and appearance of AMV could be identified. Treatment data are summarized in Table 2.

**Table 2:** Endovascular treatment success in AMV+ and AMV–.

	Total (n = 41)	AMV+ (n = 31)	AMV– (n = 10)
Treated	26/41 (63.4)	20/31 (64.5)	6/10 (60.0)
Intravenous rtPA	10/19 (52.6)	6/13 (46.2)	4/6 (66.7)
Endovascular treatment	3/6 (50.0)	3/4 (75.0)	0/2 (0.0)
Intravenous rtPA +Endovascular treatment	13/16 (81.3)	11/14 (78.6)	2/2 (100.0)

No. successfully treated/total no. of patients, (%).

## Discussion

As a principal finding of this study, we showed that the appearance of AMV indicates severe stroke and poor outcome. The fact that AMV proved to be independently associated with poor outcome after adjustment for baseline NIHSS and other influencing factors in the ordinal regression also shows that the DMV-status does have additional predicting value to the initial NIHSS. Furthermore we found AMV in two thirds of all assessed patients while no patient presented with more prominent DMV on the contralateral hemisphere, proving the principle association of DMV on the affected hemisphere and an ischemic event.

The appearance of DMV on SWI was first described by Tong et al. in 2008 who reported prominent AMV in the periphery of ischemic infarctions in children.[5] So far, this imaging pattern has not been studied systematically in adults. In contrast, the appearance of prominent cortical veins on SWI in patients with acute stroke has been reported in a few cases.[24] A pathophysiological explanation for our findings may be based on the established concept of prominent cortical veins on SWI in stroke patients. It is widely assumed that the hypointense signal within cortical veins on SWI is caused by an uncoupling between oxygen supply and demand in the hypoperfused tissue that ends up in a relative increase of deoxyhemoglobin and a relative decrease of oxyhemoglobin.[25] In case of MCA stroke, the reduced blood flow within the territory of the MCA leads to an increased oxygen extraction fraction within the tissue at risk. Hence, the concentration of deoxyhemoglobin rises within the venous system and susceptibility changes lead to the prominent hypointense signal on SWI.[26] We hypothesize that these susceptibility changes are not only visible within cortical veins but in severe cases also within the draining DMV, displaying as prominent AMV.

Even though the appearance of AMV might be interpreted as a sign of collateral flow, the clear association between poor outcome and the appearance of AMV in our study contradicts this hypothesis since high collateralization should be associated with less stroke severity and favorable outcome.[27] We therefore rather assume that the appearance of AMV reflects the increase of deoxyhemoglobin in the draining DMV above a limit of detection, indicating the decompensation of oxygen capacity, that is more likely to be reached in severe strokes.

A potential pitfall of the applied NIHSS scale in this study is that it does not differentiate between lethal cases and cases of severe stroke since both are attributed with an NIHSS score of 42.[21] However, this shortcoming of the NIHSS scale can only have influenced the results in the way that an additional consideration of lethal cases would have yielded an even higher significance of the correlation of AMV+ and outcome since lethal cases only appeared in the AMV+ group. Even though the rate of lethal cases (5 in AMV+ vs. 0 in AMV–; p = 0.08) was not significantly higher, a tendency for the correlation of AMV+ and lethal cases was observed. Accordingly, also the mRS score that differentiates between cases of severe stroke (score 5) and lethal events (score 6) was significantly different in the two groups.

Another potential bias within the assessment of NIHSS at discharge and mRS is the date of performance. Final NIHSS and mRS assessment in this study was performed at discharge. Taking into account that medium hospitalization was slightly higher in the AMV+ group (6.0 ± 3.9 days in AMV+ vs. 5.5 ± 3.1 days in AMV–; p = 0.56) and that these patients hence had more time to recover, the avoidance of this potential bias would have again resulted in an even higher significance of the correlation of AMV+ and outcome.

Interestingly, ordinal regression analysis for evaluation of AMV and clinical course of the disease assessed by the difference between NIHSS at admission and discharge did not reveal a significant correlation but only a tendency of patients with AMV having a worse clinical course compared to patients without AMV (p = 0.07). This was surprising in so far that outcome analysis revealed that patients with AMV+ had a significantly higher probability of increased mRS scores at discharge after accounting for the initial NIHSS. However, this discrepancy is likely caused by the limitations of the NIHSS score, which was originally designed as an acute score and not as an outcome score. Accordingly, clinical outcome in large stroke trials is regularly analyzed with the mRS score.[28]

Furthermore we could not find a significant difference between the groups in terms of therapy success or presence of a diffusion-perfusion mismatch. However, given the small number of cases for the different treatment groups, the probability to detect potentially existing effects was very low. Even though the measurement of thrombus length on SWI did not reach statistical significance, we found a tendency of longer thrombi in the AMV+ group. This result is in agreement with previous studies that reported thrombus extent to correlate with stroke severity and outcome.[29–31] The fact that the duration of hospitalization was not significantly different between the groups is likely biased by the fact that the therapy of the majority of patients is continued in rehabilitation facilities which was not taken into account in the current study.

A possible further limitation of our study might be that we could not assess a time dependent appearance of AMV in the group of wake up strokes. This might cause a bias, since in a former study, a case was reported in which the initial finding of prominent vessels on SWI after 6 hours disappeared after 12 hours.[32] However, it is unlikely that time dependence of AMV played a role in the current study since no significant difference between AMV– and AMV+ groups could be found between onset of symptoms and the MRI scan in the group with known symptom onset.

Finally, we recommend considering the introduced assessment of AMV in selected clinical studies. The NIHSS scoring is a standard procedure within the workflow of stroke patients and an experienced neurologist might perform an NIHSS scoring in about 4–5 minutes. However, efforts have been made to accelerate this process by the use of simplified scores [33, 34]. Since SWI is increasingly used for the detection of possible hemorrhagic transformation in acute stroke patients, no additional scanning time needs to be invested for the assessment of AMV. The AMV assessment itself only takes seconds and future studies might asses the feasibility of an integrated evaluation of AMV and its potential to save time as well as its potential to facilitate treatment decisions.

## Conclusion

The asymmetric appearance of DMV on SWI is a fast and easily evaluable parameter for the prediction of stroke severity and can be used as an additional imaging parameter in patients with acute MCA stroke.

## Supporting Information

S1 Table Interrater reliability. Interrater reliability for DMV score determined by calculation of the intraclass correlation coefficient (two-way mixed, absolute agreement).
(DOCX)

S2 Table DMV score. DMV score of each hemisphere per rater and patient.
(DOCX)

S3 Table Mann-Whitney-U Test. Comparison of NIHSS scores and mRS between AMV+ and AMV– using the Mann-Whitney-U Test.
(DOCX)

S4 Table Ordinal regression of mRS. Ordinal regression of mRS adjusted for baseline NIHSS (ungrouped), age, period of hospitalization and presence of wake up strokes. Odds ratio represents the odds of AMV+ to have a higher mRS at discharge than AMV-.
(DOCX)

S5 Table Ordinal regression analysis of ΔNIHSS. Ordinal regression analysis of ΔNIHSS adjusted for grouped NIHSS on admission, age, period of hospitalization and presence of wake up strokes. Odds ratio presents the odds of AMV+ to have a worse clinical course than AMV-.
(DOCX)

S6 Table Student t-test. Student t-test for independent samples for comparison of thrombus length, period of hospitalization age and time between onset of symptoms and MRI between AMV+ and AMV-. Positive values for difference indicate higher values in the AMV+ group.
(DOCX)
