# Supplementary material for: Asymmetry of Deep Medullary Veins on Susceptibility Weighted MRI in Patients with Acute MCA Stroke Is Associated with Poor Outcome
Source: PLoS One. 2015 Apr 7;10(4):e0120801. doi: 10.1371/journal.pone.0120801 (PMC4388537; doi:10.1371/journal.pone.0120801)
Supplement: S1 Table — Interrater reliability for DMV score determined by calculation of the intraclass correlation coefficient (two-way mixed, absolute agreement). (DOCX) [file pone.0120801.s001.docx]

**S1 Table. Interrater reliability**. Interrater reliability for DMV score determined by calculation of the intraclass correlation coefficient (two-way mixed, absolute agreement).

|  | | ICC | p-value |
| --- | --- | --- | --- |
|  | Right hemisphere | 0.88 | <0.0001 |
|  | Left hemisphere | 0.86 | <0.0001 |
|  | Difference | 0.88 | <0.0001 |
