# Supplementary material for: Asymmetry of Deep Medullary Veins on Susceptibility Weighted MRI in Patients with Acute MCA Stroke Is Associated with Poor Outcome
Source: PLoS One. 2015 Apr 7;10(4):e0120801. doi: 10.1371/journal.pone.0120801 (PMC4388537; doi:10.1371/journal.pone.0120801)
Supplement: S2 Table — DMV score of each hemisphere per rater and patient. (DOCX) [file pone.0120801.s002.docx]

**S2 Table. DMV score.** DMV score of each hemisphere per rater and patient.

|  | Rater 1 | | | Rater 2 | | |
| --- | --- | --- | --- | --- | --- | --- |
| Patient No. | Right  hemisphere | Left  hemisphere | AMV | Right  hemisphere | Left  hemisphere | AMV |
|  | 1 | 0 | + | 2 | 0 | + |
|  | 0 | 2 | + | 0 | 1 | + |
|  | 1 | 0 | + | 2 | 1 | + |
|  | 1 | 2 | + | 1 | 2 | + |
|  | 1 | 1 | - | 1 | 1 | - |
|  | 0 | 0 | - | 1 | 1 | - |
|  | 0 | 0 | - | 0 | 0 | - |
|  | 1 | 0 | + | 1 | 0 | + |
|  | 0 | 2 | + | 0 | 2 | + |
|  | 1 | 3 | + | 1 | 2 | + |
|  | 0 | 2 | + | 0 | 1 | + |
|  | 1 | 1 | - | 2 | 2 | - |
|  | 0 | 2 | + | 1 | 2 | + |
|  | 0 | 0 | - | 0 | 0 | - |
|  | 1 | 1 | - | 0 | 0 | - |
|  | 2 | 0 | + | 1 | 0 | + |
|  | 0 | 1 | + | 0 | 1 | + |
|  | 0 | 1 | + | 0 | 2 | + |
|  | 1 | 1 | - | 1 | 1 | - |
|  | 0 | 0 | - | 0 | 0 | - |
|  | 1 | 0 | + | 1 | 0 | + |
|  | 1 | 0 | + | 1 | 0 | + |
|  | 1 | 3 | + | 2 | 3 | + |
|  | 0 | 2 | + | 0 | 1 | + |
|  | 0 | 0 | - | 0 | 0 | - |
|  | 1 | 0 | + | 1 | 0 | + |
|  | 1 | 1 | - | 0 | 0 | - |
|  | 0 | 1 | + | 0 | 1 | + |
|  | 1 | 1 | - | 1 | 1 | - |
|  | 0 | 0 | - | 0 | 0 | - |
|  | 0 | 1 | + | 0 | 1 | + |
|  | 1 | 1 | - | 1 | 1 | - |
|  | 2 | 0 | + | 2 | 0 | + |
|  | 0 | 0 | - | 1 | 1 | - |
|  | 1 | 1 | - | 1 | 1 | - |
|  | 2 | 0 | + | 2 | 1 | + |
|  | 0 | 0 | - | 0 | 0 | - |
|  | 0 | 2 | + | 0 | 1 | + |
|  | 1 | 0 | + | 1 | 0 | + |
|  | 2 | 0 | + | 1 | 0 | + |
|  | 0 | 0 | - | 0 | 0 | - |
|  | 1 | 0 | + | 1 | 0 | + |
|  | 0 | 1 | + | 0 | 1 | + |
|  | 3 | 1 | + | 2 | 1 | + |
|  | 0 | 1 | + | 0 | 1 | + |
|  | 0 | 1 | + | 0 | 1 | + |
|  | 0 | 1 | + | 0 | 1 | + |
|  | 0 | 1 | + | 0 | 1 | + |
|  | 0 | 1 | + | 0 | 1 | + |
|  | 2 | 1 | + | 2 | 1 | + |
|  | 1 | 1 | - | 1 | 1 | - |
|  | 0 | 2 | + | 0 | 1 | + |
|  | 0 | 1 | + | 0 | 1 | + |
|  | 1 | 1 | - | 0 | 0 | - |
|  | 0 | 1 | + | 0 | 1 | + |
|  | 2 | 0 | + | 1 | 0 | + |
|  | 0 | 1 | + | 0 | 1 | + |
|  | 0 | 1 | + | 0 | 1 | + |
|  | 1 | 1 | - | 1 | 1 | - |
|  | 0 | 0 | - | 0 | 0 | - |
|  | 2 | 0 | + | 2 | 1 | + |
|  | 2 | 0 | + | 2 | 0 | + |
|  | 0 | 1 | + | 0 | 1 | + |
|  | 1 | 1 | - | 1 | 1 | - |
|  | 0 | 1 | + | 0 | 1 | + |
|  | 1 | 1 | - | 1 | 1 | - |
|  | 1 | 0 | + | 1 | 0 | + |
|  | 1 | 3 | + | 0 | 2 | + |
|  | 0 | 1 | + | 0 | 1 | + |
|  | 0 | 1 | + | 0 | 1 | + |
|  | 1 | 0 | + | 1 | 0 | + |
|  | 1 | 1 | - | 1 | 1 | - |
|  | 0 | 0 | - | 0 | 0 | - |
|  | 0 | 2 | + | 0 | 1 | + |
|  | 0 | 0 | - | 0 | 0 | - |
|  | 1 | 2 | + | 0 | 1 | + |
|  | 1 | 1 | - | 0 | 0 | - |
|  | 1 | 2 | + | 0 | 1 | + |
|  | 0 | 1 | + | 0 | 1 | + |
|  | 1 | 1 | - | 0 | 0 | - |
|  | 0 | 1 | + | 0 | 2 | + |
|  | 0 | 0 | - | 0 | 0 | - |
|  | 0 | 0 | - | 0 | 0 | - |
|  | 0 | 2 | + | 0 | 1 | + |
|  | 0 | 0 | - | 0 | 0 | - |
|  | 0 | 2 | + | 0 | 2 | + |
