# Supplementary material for: Asymmetry of Deep Medullary Veins on Susceptibility Weighted MRI in Patients with Acute MCA Stroke Is Associated with Poor Outcome
Source: PLoS One. 2015 Apr 7;10(4):e0120801. doi: 10.1371/journal.pone.0120801 (PMC4388537; doi:10.1371/journal.pone.0120801)
Supplement: S3 Table — Comparison of NIHSS scores and mRS between AMV+ and AMV– using the Mann-Whitney-U Test. (DOCX) [file pone.0120801.s003.docx]

**S3 Table. Mann-Whitney-U Test.** Comparison of NIHSS scores and mRS between AMV+ and AMV– using the Mann-Whitney-U Test.

|  | | | | |  |
| --- | --- | --- | --- | --- | --- |
| Variable | AMV | N | Mean Rank | Sum of Ranks | p-value |
| NIHSS on admission | + | 55 | 49.99 | 2749.50 |  |
|  | – | 31 | 31.98 | 991.50 |  |
|  | Total | 86 |  |  | 0.001 |
| NIHSS at discharge | + | 55 | 49.12 | 2701.50 |  |
|  | – | 31 | 33.53 | 1039.50 |  |
|  | Total | 86 |  |  | 0.005 |
| mRS | + | 55 | 49.98 | 2749.00 |  |
|  | – | 31 | 32.00 | 992.00 |  |
|  | Total | 86 |  |  | 0.001 |
