# Supplementary material for: Asymmetry of Deep Medullary Veins on Susceptibility Weighted MRI in Patients with Acute MCA Stroke Is Associated with Poor Outcome
Source: PLoS One. 2015 Apr 7;10(4):e0120801. doi: 10.1371/journal.pone.0120801 (PMC4388537; doi:10.1371/journal.pone.0120801)
Supplement: S4 Table — Ordinal regression of mRS adjusted for baseline NIHSS (ungrouped), age, period of hospitalization and presence of wake up strokes. Odds ratio represents the odds of AMV+ to have a higher mRS at discharge than AMV-. (DOCX) [file pone.0120801.s004.docx]

**S4 Table. Ordinal regression of mRS**. Ordinal regression of mRS adjusted for baseline NIHSS (ungrouped), age, period of hospitalization and presence of wake up strokes. Odds ratio represents the odds of AMV+ to have a higher mRS at discharge than AMV-.

|  | Odds ratio | p-value | 95% Confidence Interval | |
| --- | --- | --- | --- | --- |
|  |  |  | Lower | Upper |
| Higher mRS for AMV+ | 3.19 | 0.016 | 1.24 | 8.21 |
