# Supplementary material for: Asymmetry of Deep Medullary Veins on Susceptibility Weighted MRI in Patients with Acute MCA Stroke Is Associated with Poor Outcome
Source: PLoS One. 2015 Apr 7;10(4):e0120801. doi: 10.1371/journal.pone.0120801 (PMC4388537; doi:10.1371/journal.pone.0120801)
Supplement: S5 Table — Ordinal regression analysis of ΔNIHSS adjusted for grouped NIHSS on admission, age, period of hospitalization and presence of wake up strokes. Odds ratio presents the odds of AMV+ to have a worse clinical course than AMV-. (DOCX) [file pone.0120801.s005.docx]

**S5 Table. Ordinal regression analysis of ΔNIHSS.** Ordinal regression analysis of ΔNIHSS adjusted for grouped NIHSS on admission, age, period of hospitalization and presence of wake up strokes. Odds ratio presents the odds of AMV+ to have a worse clinical course than AMV-.

|  | Odds ratio | p-value | 95% Confidence Interval | |
| --- | --- | --- | --- | --- |
|  |  |  | Lower | Upper |
| Lower ΔNIHSS for AMV+ | 0.43 | 0.07 | 0.18 | 1.07 |
