# Supplementary material for: Asymmetry of Deep Medullary Veins on Susceptibility Weighted MRI in Patients with Acute MCA Stroke Is Associated with Poor Outcome
Source: PLoS One. 2015 Apr 7;10(4):e0120801. doi: 10.1371/journal.pone.0120801 (PMC4388537; doi:10.1371/journal.pone.0120801)
Supplement: S6 Table — Student t-test for independent samples for comparison of thrombus length, period of hospitalization age and time between onset of symptoms and MRI between AMV+ and AMV-. Positive values for difference indicate higher values in the AMV+ group. (DOCX) [file pone.0120801.s006.docx]

**S6 Table**. **Student t-test.** Student t-test for independent samples for comparison of thrombus length, period of hospitalization age and time between onset of symptoms and MRI between AMV+ and AMV-. Positive values for difference indicate higher values in the AMV+ group.

| Variable | | Student  t-statistic | Degrees of freedom | p-value | Mean Difference | 95% Confidence Interval of the Difference | |
| --- | --- | --- | --- | --- | --- | --- | --- |
|  |  |  |  |  |  | Lower | Upper |
| Age (y) |  | -0.22 | 84 | 0.98 | -0.08 | -7.03 | 6.88 |
| Time between onset of symptoms and MRI (hs) |  | -0.39 | 25 | 0.70 | -0.28 | -1.76 | 1.19 |
| Period of hospitalization (days) |  | 0.59 | 84 | 0.56 | 0.49 | -1.15 | 2.13 |
| Thrombus length (mm) |  | 2.93 | 63.60 | 0.05 | 3.95 | 1.26 | 6.65 |
